# Cu_**4**_ Cluster Doped Monolayer MoS_2_ for CO Oxidation

**DOI:** 10.1038/srep11230

**Published:** 2015-06-08

**Authors:** Z. W. Chen, J. M. Yan, W. T. Zheng, Q. Jiang

**Affiliations:** 1 Key Laboratory of Automobile Materials, Ministry of Education, and School of Materials Science and Engineering, Jilin University, Changchun 130022, China

## Abstract

The catalytic oxidation of CO molecule on a thermodynamically stable Cu_4_ cluster doped MoS_2_ monolayer is investigated by density functional theory (DFT) where the reaction proceeds in a new formation order of COOOCO* (O_2_* + 2CO* → COOOCO*), OCO* (COOOCO* → CO_2_ + OCO*), and CO_2_ (OCO* → CO_2_) desorption with the corresponding reaction barrier values of 0.220 eV, 0.370 eV and 0.119 eV, respectively. Therein, the rate-determining step is the second one. This low barrier indicates high activity of this system where CO oxidation could be realized at room temperature (even lower). As a result, the Cu_4_ doped MoS_2_ could be a candidate for CO oxidation with lower cost and higher activity without poisoning and corrosion problems.

With the rapid development of industry, the amount of carbon monoxide (CO) emission, resulted from automobiles, industrial processes and so on, drastically increases. Currently, the most effective way in reducing CO is CO oxidation where the decrease of the energy barrier values (*E*_bar_) with effective catalysts is most technically concerned^1^,^2^,^3^. Besides the element metals, such as Pt^1^,^4^,^5^, Pd^6^, Ru^7^, Au^8^, etc., alloys, oxides and others are also selected as catalysts^9^,^10^,^11^,^12^,^13^,^14^, where the higher activity, the higher oxidation and poison resistances, and the lower cost, are important indexes.

Single atomic metal catalyst anchored on appropriate support has the maximal usage of metal atoms and great potential to achieve high activity and selectivity^15^. The difficulty of this usage is its aggregation into big clusters on the support due to the high surface free energy of metal atoms. Supported metal clusters are an alternative choice. For instance, MgO supported Au_8_ cluster and Fe_3_O_4_ supported Pd_n_ clusters show good catalytic activities for CO oxidation^16^,^17^ where the catalytic activities of supported metal clusters are strongly size-dependent and shape-dependent^18^,^19^.

Copper-based catalysts or catalyst promoters have attracted persistent interests because of their wide applications in a variety of industrial processes^10^,^20^,^21^,^22^,^23^. For instance, copper-based nanoparticles supported on oxide substrates show superior catalysis for low temperature CO oxidation and resistance against water contamination^24^,^25^. The Cu embedded in graphene has been proved to be a good candidate for CO oxidation with lower cost and higher activity^26^. However, easy oxidation of Cu atoms leads to low service life of copper-based catalysts.

Graphene is a promising matrix supporting metal atoms to catalyze CO oxidation due to its outstanding electrical, mechanical and thermal properties^27^,^28^,^29^,^30^ with large surface/volume ratio. However, the thermal stability and chemical reactivity issues associated with graphene may hinder its applications^31^,^32^ due to weaker anchoring ability of atoms and clusters and less controlling ability of the shapes of atoms and clusters resulted from graphene’s single-layer structure^33^,^34^. The monolayer MoS_2_ has a similar two-dimensional (2D) structure of graphene and is inert due to the absence of dangling bonds at the basal planes terminated by S atoms^35^. Differing from graphene, MoS_2_ monolayer consisting of S-Mo-S sandwich layer could well fix and regulate the morphology of clusters as expected. It could be an alternative of graphene and has been acted as the catalyst for the hydrogen evolution reaction (HER) and CO oxidation^36^,^37^,^38^,^39^.

In this work, the tetrahedral structure of four Cu atoms (Cu_4_) embedded in monolayer MoS_2_ is carried out through first principles calculations where one Mo atom and three S atoms in the monolayer MoS_2_ are substituted by Cu_4_ since the unique triangular active site of Cu_3_ has been identified as a crucial role for CO oxidation^8^. This structure is stable due to the strong chemical bonds among Cu_4_ and monolayer MoS_2_^25^ while the triangular Cu_3_ active site is acted for CO oxidation by adsorbing O_2_ and more CO molecules and the Cu_4_ cluster completely inserts into the sandwich of MoS_2_. We have found a new OCO* intermediate state (MS) with a small *E*_bar_ of 0.370 eV on Cu_4_ cluster during the CO oxidation process. Our calculations suggest that the Cu_4_ cluster embedded in a monolayer MoS_2_ is a good candidate for CO oxidation.

## Results and discussion

**Figure 1:**
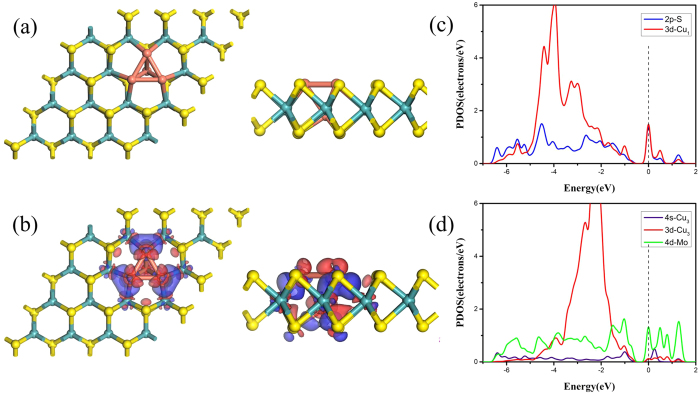
(**a**) Top and side views of the geometric configurations of Cu_4_-doped monolayer MoS_2_. The yellow, blue, and orange balls represent the S, Mo, and Cu atoms. The surface three Cu atoms called Cu_3_, the other Cu called Cu_1_. (**b**) The charge density difference of Cu_4_-doped MoS_2_, the blue and red regions represent the electron accumulation and loss, respectively. (**c**), (**d**) show the spin-polarized partial density of states (PDOS). In (**c**), the red and blue lines indicate the orbitals of Cu_1_-3*d* and S-2*p*, respectively. In (**d**), the blue, red and green lines indicate the orbitals of Cu_3_-4*s*, Cu_3_-3*d* and Mo-4*d*, respectively. The Fermi level is set to zero.

The experimental fabrication of the Cu_4_ doped MoS_2_ could be intricate since there are two different doping sites on monolayer MoS_2_, Mo vacancy and S vacancy^40^,^41^. It is known that Re atoms and Co atoms occupying Mo sites in monolayer MoS_2_ have been synthesized by the chemical vapor transport (CVT) and chemical vapor deposition (CVD) method, respectively^42^,^43^. Since the value of Pauling electronegativity of Cu (1.90) is almost the same of Re (1.90) and Co (1.88), the Cu atom could substitute Mo atom in the MoS_2_ by means of CVT or CVD, which has been proved to be feasible through the density functional calculations^25^. Then, the S vacancies are prepared by low-energy argon sputtering or electron irradiation. Last, the Cu_3_ atoms are embedded into these vacant sites through the physical vapor deposition. The corresponding structure of Cu_4_ doped MoS_2_ is shown in Fig. 1(a) where one Cu atom substituting the Mo atom is denoted as Cu_1_, and the other three Cu atoms replacing three S atoms on the surface of monolayer MoS_2_ are named as Cu_3_. The flat triangular Cu_3_ active site on the surface plays a vital role for CO oxidation^8^. The Cu_3_ atoms and surface S atoms are at the same plane, making it less active to be oxidized than the metal atoms above the graphene surface. The bond lengths of Cu_3_-Cu_3_ and Cu_3_-Cu_1_ are 2.53 Å and 2.57 Å, being almost the same of Cu-Cu (2.55 Å) in Cu bulk. The bond lengths of Cu_3_-Mo and Cu_1_-S are 2.66 Å and 2.28 Å. The latter is shorter than the bond length of Cu-S of 2.41 Å in Cu-doped MoS_2_^25^. Thus, Cu_1_-S bond is stronger than Cu-S bond. From Hirshfeld charge analysis, the electron transfer of Cu_1_ and Cu_3_ are 0.146 *e* and 0.076 *e*, resp*e*ctively. The direction of electron transfer is in agreement with the values of Pauling electronegativity of Cu (1.90), S (2.58) and Mo (2.16)^44^. There are about 0.374 *e* transf*e*r from the Cu_4_ cluster to monolayer MoS_2_. The electron transfer can also be verified by the charge density difference (CDD) for Cu_4_ doped MoS_2_. As shown in Fig. 1(b), the blue and red regions represent the areas of electron accumulation and depletion, respectively. Obviously, different electron affinities of Cu, S and Mo determine the electron distribution. The pronounced charge density redistribution on the Cu_3_-Mo bonds and Cu_1_-S bonds [Fig. 1(b)] indicates stronger interaction between Cu_4_ and MoS_2_.

The partial density of states (PDOS) projected on the 3*d* orbitals of Cu_1_, the 2*p* orbitals of its neighboring S, 3*d* orbitals and 4*s* orbitals of Cu_3_ and 4*d* orbitals of its neighboring Mo are plotted, as shown in Fig. 1(c),(d). The strong interaction between Cu_1_ and S can be further confirmed by the PDOS in Fig. 1(c) where significant hybridization between Cu_1_-3*d* and the a*d*jacent S-2*p* is present, denoting the stability of the system. Furthermore, the hybridization between 4*s* and 3*d* orbitals of Cu_3_ and the adjacent Mo-4*d* is also found in Fig. 1(d).

**Figure 2:**
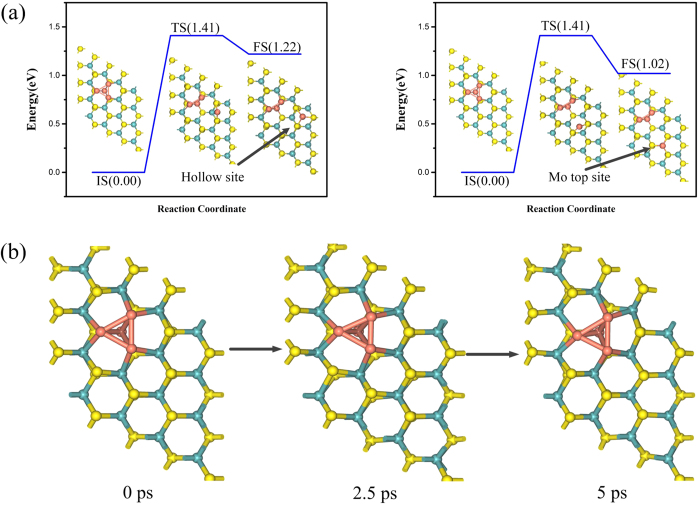
(**a**) The diffusion paths of the Cu atom from the S vacancy to its neighboring hollow site of the S-Mo-S hexagonal ring (left) and Mo top site (right), including the initial state (IS), transition state (TS), and final state (FS). The values (in eV) show all energies are given with respect to the reference energy. (**b**) The three structures from MD simulation in 5 ps at the temperature of 500 K are shown.

To gain more insight into the stability of Cu_4_ doped MoS_2_, we also calculated the *E*_b_ of Cu_4_ doped MoS_2_, being 3.262 eV, showing strong interaction between Cu_4_ cluster and its neighboring S and Mo atoms. For the possible diffusion problem of Cu atom, we computed the energy barrier and reaction energy of Cu diffusion. Since the Cu_1_ strongly bonds with three S atoms and it is in the middle layer of the Cu_4_ doped MoS_2_, the diffusion of the Cu_1_ atom is difficult. Therefore, only the surface Cu_3_ atoms are considered here. Because all three Cu_3_ atoms are the same in symmetry, we study only one Cu_3_ atom diffusing to the neighboring hollow site or Mo top site [Fig. 2(a)]. The diffusion energy barriers for the two cases are the same with a value of 1.41 eV and the reaction energies are 1.02 eV and 1.22 eV, respectively. Considering the high diffusion barriers and the endothermic reactions, the diffusion of Cu_3_ is absent and the Cu_4_ doped MoS_2_ system thus is an energetically stable structure.

To further prove the stability of the system, first principle molecular dynamics (MD) simulation at a constant temperature of *T* = 500 K in the NVT ensemble (i.e., constant particle number, volume and temperature condition) has been carried out for 5 *ps* with the time step of 1 *fs*. Three structures from MD calculation are present in Fig. 2(b). It is found that Cu_4_ cluster is fixed in the vacancies of MoS_2_ and the Cu atoms are located at the original sites after 5000 dynamics steps at 500 K. Thus, the stability of the studied Cu_4_ doped MoS_2_ system at room temperature is expected.

**Figure 3:**
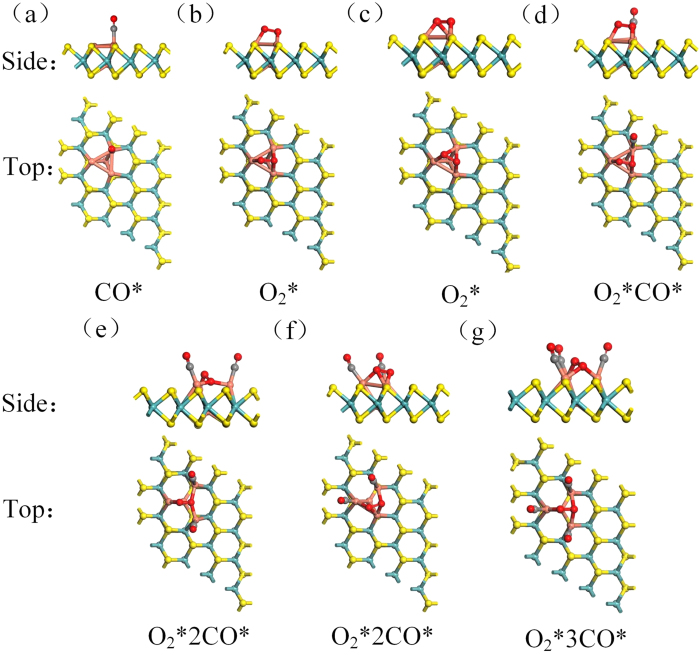
Top and side views of the geometric configurations of adsorption sites of O_2_ and CO. yellow, blue, orange, red, gray balls represent the S, Mo, Cu, O, C atoms.

The adsorption process of O_2_ molecule on Cu_4_ doped MoS_2_ is considered for possible side-on and end-on configurations. With the former configuration, two found adsorption structures of O_2_ molecule are shown in Fig. 3(b), which are defined as t-h-b and b-h-b with *E*_ad-O2_ values of −1.743 eV and −1.749 eV (Table 1). With the latter configuration, there are three adsorption structures. The related *E*_ad-O2_ values are −0.867 eV,−0.868 eV and −0.602 eV on hollow, bridge and top sites. The above results imply that the O_2_ molecule prefers the side-on configurations, which gets 0.399 *e* and 0.420 *e* respectively.

**Figure 4:**
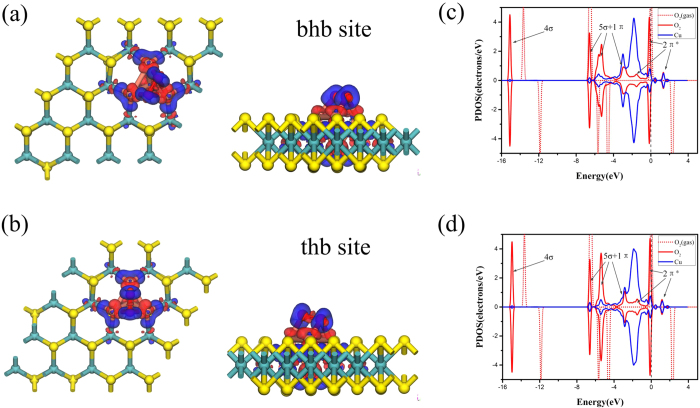
Top and side views of the O_2_ adsorption on bhb (bridge-hollow-bridge) site and thb (top-hollow-bridge) site of Cu_4_-doped MoS_2_ with the charge density difference are shown in (**a**) and (**b**), respectively. The blue and red regions represent the election accumulation and loss. (**c**) and (**d**) show corresponding PDOS. The red and blue lines indicate the orbitals of the adsorbed O_2_ and Cu atom. The red dotted line and the vertical black dotted line denote the orbitals of O_2_ molecule (gas) and the Fermi level.

Being consistent with the Hirshfeld charge analysis, the PDOS of O_2_ molecule, adsorbed O_2_ and Cu atom are shown in Fig. 4(c). All orbitals of O_2_ are labeled while the 2π* anti-bond orbital is half filled, which is in agreement with literature data^45^. When O_2_ is adsorbed on Cu_3_, significant charge transfers (0.420 *e* and 0.399 *e*) from Cu_4_ doped MoS_2_ to O_2_ are found, which occupy the initial empty component of the O_2_-2π* orbitals and lead to the elongation of the O-O bond from 1.224 Å to 1.484 Å and 1.467 Å respectively. The hybridization between Cu atom and O_2_-2π* orbitals is located near Fermi level.

For CO oxidation reaction, the adsorption of CO and CO_2_ also should be considered. In this system, the *E*_ad-CO_ values are −1.105 eV on top site,−0.990 eV on bridge site and −0.957 eV on hollow site, respectively. Thus, CO prefers adsorbing on the top site, as illustrated in Fig. 3(a). Since *E*_ad-O2_ (−1.749 eV) is stronger than *E*_ad-CO_ (−1.105 eV), O_2_ is preferentially adsorbed on the hollow site, which indicates there is no CO poisoning problem. And the *E*_ad-CO2_ value is −0.140 eV, which proves that the CO_2_ is easy to leave the surface. Note that as long as the first O_2_ is adsorbed on the hollow site, sites for other O_2_ adsorptions are absent. Because the adsorption energy value of two O_2_ on the catalyst is −1.709 eV, which is weaker than that of only one O_2_. As a result, the Cu_4_ doped MoS_2_ without poisoning and oxidation problems could be a good catalyst with a long cycle life. To further more comprehensively understand the adsorption of O_2_ and CO on the catalyst, the other sites near the Cu_4_ cluster are considered. Because MoS_2_ surface is inert at the basal planes terminated by S atoms, both *E*_ad-O2_ value and *E*_ad-CO_ value are about −0.1 eV, which imply that O_2_ and CO only are adsorbed on Cu_4_ cluster of the catalyst.

**Figure 5:**
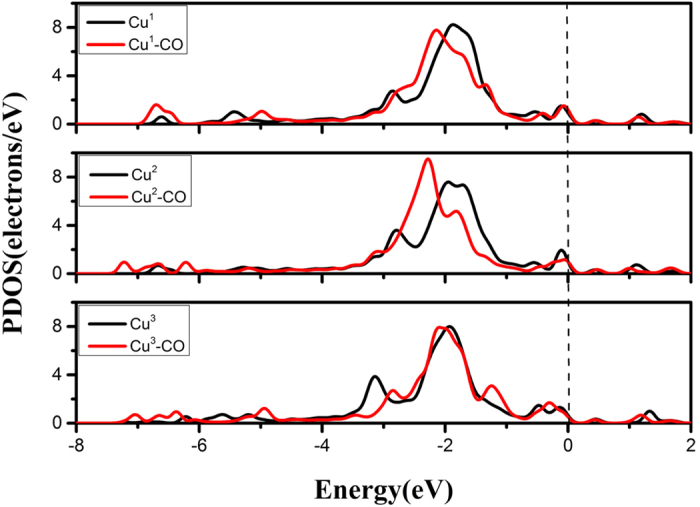
The PDOS of Cu sites without CO (Cu^n^) and with CO (Cu^n^-CO). The black and red lines indicate the orbitals of Cu^n^ and Cu^n^-CO, respectively. The Fermi level is set to zero and n is the number of CO molecule.

All configurations and their *E*_ad-nCO_ values are given in Table 1. For the co-adsorption of O_2_ + nCO, the *E*_ad-nCO_ are −0.401 eV,−0.350 eV and −0.186 eV respectively for n = 1, 2, 3. The corresponding PDOS of Cu^n^ and Cu^n^-CO are shown in Fig. 5. When n = 1 and 2, the orbitals below Femi level are away from the Femi level, denoting more stable states. If n = 3, however, the orbitals change less, denoting weaker interaction of the third CO with Cu atom. To simplify the latter discussion, we neglect the adsorption of the third CO on Cu_4_ cluster in the following.

Now we begin to consider CO oxidation on Cu_4_ doped MoS_2_. It is well known that there are two mechanisms for CO oxidation: Langmuir-Hinshelwood (LH) mechanism and Eley-Rideal (ER) mechanism. The former involves all the reacting intermediates on the surface, whereas the latter does species from the direct reaction with a surface intermediate^46^,^47^. Both will be discussed in details in the following.

**Figure 6:**
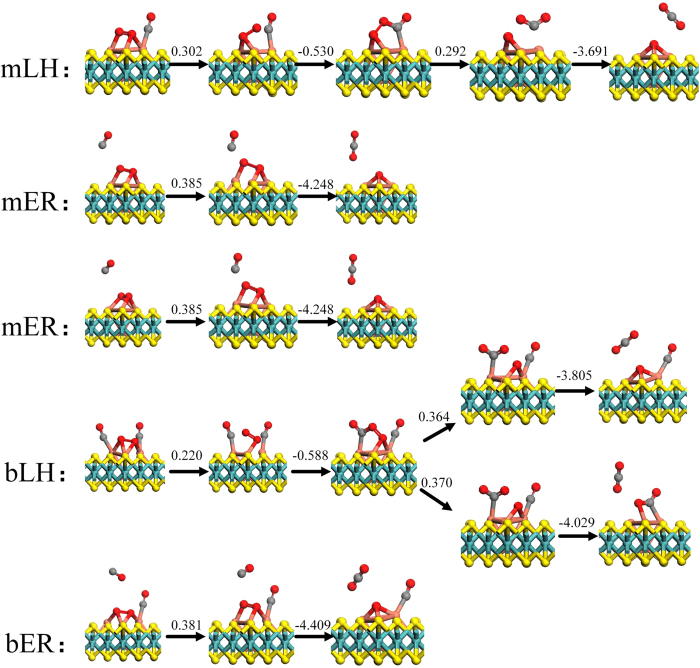
The reaction paths (side views) of the mLH, mER, bLH and bER of the first CO_2_ release. m: monomolecular (only one CO molecule); b: bimolecular (two CO molecules); IS: initial state; MS: intermediate state; TS: transition state; FS: final state. The values are the relative energies and in unit of eV.

Firstly, the LH mechanism of the one CO co-adsorption with O_2_ molecule is considered, which is denoted as mLH shown in Fig. 6 (top views in Fig. S1). The O atom in O_2_ molecule approaches the CO molecule and bonds with the C atom of the CO molecule to form the OCOO* intermediate state (MS1 in Fig. 6) with the *E*_bar_ = 0.302 eV. Then, the O-O bond breaks and CO_2_ molecule is released from the catalyst with *E*_bar_ = 0.292 eV. Therein, the rate-determining step for the mLH mechanism is the formation of OCOO* intermediate (TS1 in Fig. 6). On the other hand, for mER mechanism, the first un-adsorbed CO molecule directly reacts with the activated O_2_ molecule. Due to the presence of two adsorption configurations of O_2_ molecule, there are two kinds of mER mechanisms in the reaction path (mER in Fig. 6). The migration of CO molecule toward the pre-adsorbed O_2_ molecule is determined as the rate-determining step with *E*_bar_ = 0.385 eV (TS3 and TS4 in Fig. 6).

When the O_2_ molecule and first CO molecule have been adsorbed on the triangular Cu_3_ active site, the second CO molecule can be further adsorbed, which reacts with the adsorbed O_2_. We assume that the reaction could follow bLH mechanism or bER mechanism, we define b denoting the case where two CO molecules are involved in the reaction path. In the bLH mechanism, the adsorption structures of the two CO molecules and O_2_ molecule are shown in Fig. 3(e). The initial structure of Fig. 3(f) goes to the OC-OCOO* intermediate state (MS2 in Fig. 6) with *E*_bar_ = 0.220 eV {while another [Fig. 3(e)] does not}, where the O-O bond is broken up and two metastable states are present (FS4 and FS5 in Fig. 6). Their *E*_bar_ values are 0.364 eV and 0.370 eV, respectively. Although both values are pretty much the same, the product of FS5, OCO*, releases 0.22 eV more energy for the formation of first CO_2_ than that of FS4 while OCO* is beneficial for the next oxidation reaction. As a result, FS5 tends to happen relative to FS4. In the case of bER mechanism (Fig. 6), *E*_bar_ = 0.381 eV for the release of the first CO_2_.

After releasing the first CO_2_, the following structures are shown in Fig. 6(FS1~FS6). In addition to OCO* (FS5), others have one O atom on the triangular Cu_3_ site. The O atom needs larger *E*_bar_ to form CO_2_ with CO due to the strong interaction between the Cu_3_ atoms and O atom, as shown in Fig. S2. However, the formation of CO_2_ from the OCO* intermediate state has the smallest *E*_bar_ (0.119 eV) to escape from the surface of the catalyst, implying desorption of the second CO_2_.

**Figure 7:**
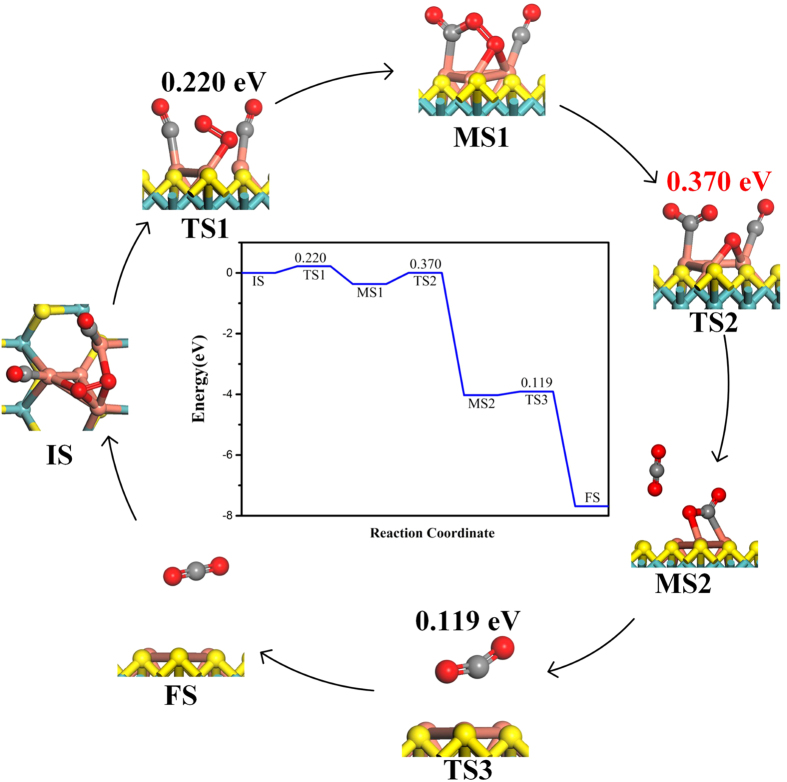


The above results show that the LH mechanism is better than the corresponding ER mechanism as the O_2_ molecule itself is not activated enough without the cooperative adsorption of CO. Then, the co-adsorbed CO molecules affect the *E*_bar_ while the rate-determining step changes from the first step to the second one. The *E*_bar_ of ER mechanism is affected by the adsorbed number of CO molecule too. Last, it should be noted that in the bLH mechanism, the OCO* (FS5 in Fig. 6) is found as the last product which is particularly favorable for the release of the second CO_2_ with *E*_bar_ = 0.119 eV. As a result, the most optimal reaction path is given in Fig. 7. Among three *E*_bar_ values in the reaction path, the largest *E*_bar_ value is 0.370 eV where the rate-determining step is the formation of the OCO* intermediate state. Thus, no matter from the point of view of dynamics or thermodynamics, the reaction path (Fig. 7) is the most optimal process. Because of the complexity and diversity of the experimental conditions, there are a few other reaction paths in CO oxidation reaction. All kinds of reaction paths and their *E*_bar_ values are shown in Fig. S3 and Fig. S4.

Now we compare the corresponding *E*_bar_ values at the rate-determining reaction step for our system and related systems which are listed in Table 2. As shown in Table 2, Zn-embedded graphene and Au-embedded graphene have smaller *E*_bar_ than this system. However, it is noteworthy that our system is more stable than Zn-embedded graphene while Au-embedded graphene has CO poisoning problem, thus their applications are limited. Thus, the overall performance of Cu_4_ doped MoS_2_ for the CO oxidation should be the best among the considered systems.

Under the biggest doping concentration, Cu_4_ doped 3 × 3 monolayer MoS_2_ supercell is also considered for CO oxidation. For CO and O_2_ adsorption, their adsorption energy values are shown in Table 1 where the both supercells have almost the same values. The stability of 3 × 3 supercell is also determined, as shown in Fig. S5. The results show that even Cu_4_ doped monolayer MoS_2_ has the biggest doping concentration, it still possesses good catalytic activity and stability for CO oxidation as our results from the 4 × 4 supercell studied above.

In summary, our comprehensive DFT studies of CO oxidation on Cu_4_ doped monolayer MoS_2_ suggest that the protruded triangular Cu_3_ site is the main active site for CO catalytic oxidation while the number of CO adsorbed molecule produces a significant effect on the energy barriers of the CO oxidation reaction. During the reaction, an OCO* intermediate state is found, which leads to the energy barrier of CO oxidation of 0.370 eV. As a result, the Cu_4_ doped monolayer MoS_2_ with outstanding catalytic activity without poisoning and oxidation problems could be a good candidate for CO oxidation with low cost and high activity.

## Methods

In this work, all calculations are performed using the spin-unrestricted density functional theory (DFT) as implemented in the DMol^3^ code^48^. Exchange-correlation functions are taken as a generalized gradient approximation (GGA) with Perdew-Wang correlation (PWC)^49^. DFT semi-core pseudo potentials (DSPPs) core treatment^50^ is implemented for relativistic effects, which replaces core electrons by a single effective potential. In addition, double numerical plus polarization (DNP) is chosen as the basis set and the quality of orbital cutoff is fine. The convergence criteria of the geometrical optimization are set to be 1.0 × 10^−5^ hartree for the energy change, 2.0 × 10^−3^ hartree/Å for the gradient, and 5.0 × 10^−3^ Å for the displacement, respectively. The smearing parameter is set to be 0.005 hartree in the geometric optimization. For transition states (TS) searching, the calculation firstly performs a linear synchronous transit (LST)^51^ maximum, which is followed by an energy minimization in directions conjugating to the reaction pathway. TS approximation obtained via LST/optimization is then used to perform a quadratic synchronous transit (QST)^51^ maximization to find more accurate transitional states. The convergence tolerance of the root mean square (RMS) force is 2.0 × 10^−3^ hartree/Å and the maximum number for QST step is set as 10. In the simulation, three-dimensional periodic boundary conditions are taken. The simulation cell consists of a 4 × 4 monolayer MoS_2_ supercell with a vacuum width of 18 Å, which leads to negligible interactions between the system and their mirror images. In order to prove the effect of doping concentration, 3 × 3 monolayer MoS_2_ supercell is also considered. For geometric optimization and the search for the transition state (TS), the Brillouin zone integration is performed with 3 × 3 × 1 k-point sampling. After structure relaxations, the density of states (DOS) are calculated with a finer k-point grid of 15 × 15 × 1 to achieve high accuracy, and the empty bands are chosen as 12. Concerning with the properties of charge transfers, atom charges would be calculated via the Hirshfeld population analysis^52^,^53^.

In the above system, the binding energy value *E*_b_ (Cu_4_) is defined as^34^,


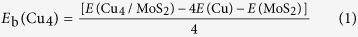


where *E*(MoS_2_), *E*(Cu) and *E*(Cu_4_/MoS_2_) are the total energies of the monolayer MoS_2_ with three S vacancies and a Mo vacancy, the free Cu atom, and the Cu_4_ doped MoS_2_, respectively.

For one molecule (CO, O_2_, CO_2_) adsorbed on catalyst, the adsorption energy values of *E*_ad-M_ (the subscript M denotes the corresponding molecule) are determined by,


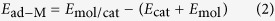


where *E*_mol/cat_, *E*_cat_ and *E*_mol_ are total energies of the molecules/catalytic system, the isolate catalyst, and the molecule.

For several molecules (CO and O_2_) co-adsorbed on the catalyst, the adsorption energy value *E*_ad-nCO_ is determined by,


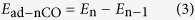



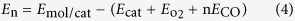


where 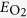 and 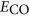 are total energy values of O_2_ and CO, and n represents the number of CO.

The van der Waals interaction is taken into account by using DFT-D functional in Dmol^3^. The *E*_ad-CO_ and *E*_ad-O2_ values recalculated are now −1.226 eV and −1.842 eV respectively, which are a little stronger than the values of *E*_ad-CO_ = −1.105 eV and *E*_ad-O2 _= −1.749 eV calculated without the consideration of the van der Waals interaction. About the reaction processes, we study the most optimal path and the rate-determining steps of all reaction paths. The reaction barrier values of O_2_* + 2CO* → COOOCO*, COOOCO* → CO_2_ + OCO* and OCO* → CO_2_ are 0.224 eV, 0.403 eV and 0.131 eV respectively, which are almost the same as the corresponding reaction barrier values of 0.220 eV, 0.370 eV and 0.119 eV without the consideration of the van der Waals interaction. And the reaction barrier values of the rate-determining steps of other reaction paths are 0.533 eV, 0.577 eV, 0.457 eV, 0.421 eV, 0.524 eV and 0.465 eV respectively. As a result, the most optimal reaction path of O_2_* + 2CO* → COOOCO*, COOOCO* → CO_2_ + OCO* and OCO* → CO_2_ for the path of CO oxidation remains correct.

## Additional Information

**How to cite this article**: Chen, Z. W. *et al.* Cu4 Cluster Doped Monolayer MoS2 for CO Oxidation. *Sci. Rep.* **5**, 11230; doi: 10.1038/srep11230 (2015).

## Supplementary Material

Supplementary Information

## Figures and Tables

**Table 1:** **The adsorption energies** ***E***_**ad-M**_ **of the O**_**2**_ **or CO,** ***E***_**ad-nCO**_ **values of 4 × 4 and 3 × 3 supercell of the Cu**_**4**_ **doped monolayer MoS**_**2**_.

**Supercell**	**4 × 4**	**3 × 3**
*E*_ad-CO_	−1.105 eV	−1.095 eV
*E*_ad-O2_	−1.743 eV (−1.749 eV)	−1.731 eV (−1.741 eV)
*E*_ad-1CO_	−0.401 eV	−0.420 eV
*E*_ad-2CO_	−0.350 eV (−0.315 eV)	−0.351`eV (−0.351 eV)
*E*_ad-3CO_	−0.186 eV	−0.235 eV

The negative sign means exothermic process. The values in parentheses represent *E*_ad_ values of another configuration.

**Table 2:** **The** ***E***_**bar**_ **values of rate-determining reaction of different systems.**

**Systems**	***E***_**bar**_ **(eV)**	**Reference**
Cu_4_-doped monolayer MoS_2_	0.37	This work
Cu-doped graphene	0.59	26
Fe-doped monolayer MoS_2_	0.51	38
Au-doped *h*-BN monolayer	0.47	32
graphene/Pt (1 1 1)	0.51	5
Zn-embedded graphene	0.26	29
Au-embedded graphene	0.31	30
