# Supplementary material for: Cu4 Cluster Doped Monolayer MoS2 for CO Oxidation
Source: Sci Rep. 2015 Jun 8;5:11230. doi: 10.1038/srep11230 (PMC4459236; doi:10.1038/srep11230)
Supplement: Supplementary Information [file srep11230-s1.docx]

**Supporting Information**

***for***

**Cu_4_ Cluster Doped Monolayer MoS_2_ for CO Oxidation**

Z. W. Chen, J. M. Yan, W. T. Zheng, Q. Jiang^[[1]](#footnote-1)^*

*Key Laboratory of Automobile Materials, Ministry of Education, and School of Materials Science and Engineering, Jilin University, Changchun 130022, China*

**Figure S1.** The reaction paths (top views) of the mLH, mER, bLH and bER of the first CO_2_ release. m: monomolecular (only one CO molecule); b: bimolecular (two CO molecules); IS: initial state; MS: intermediate state; TS: transition state; FS: final state. The values are the relative energies and in unit of eV.

**
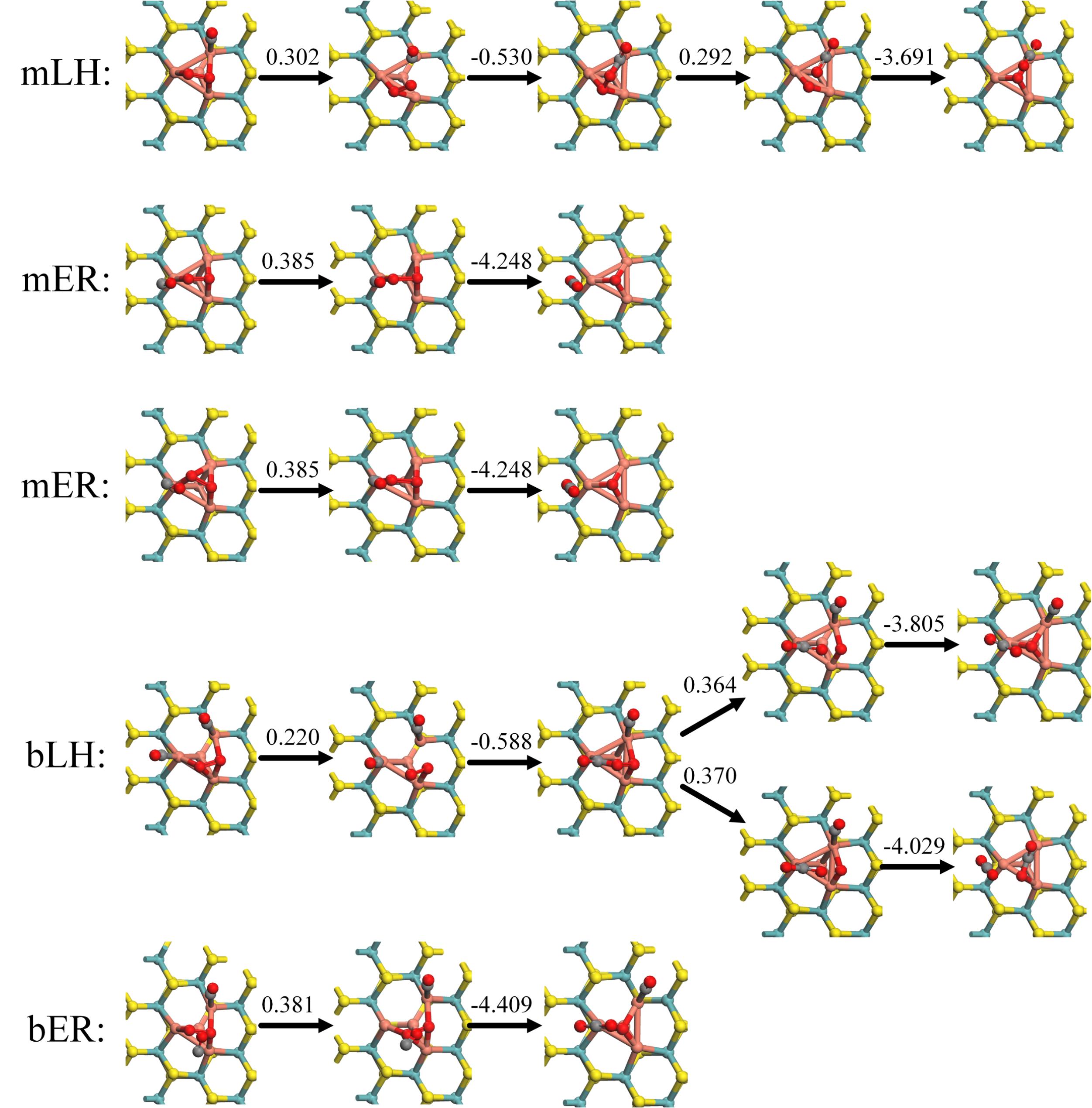
**

**Figure S2.**The reaction paths of the mLH, mER, bLH, bER, tLH and tER of the second CO_2_ release. m: monomolecular (only one CO molecule); b: bimolecular (two CO molecules); t: trimolecular (three CO molecules); IS: initial state; MS: intermediate state; TS: transition state; FS: final state. The values indicated the corresponding energy barriers.

**
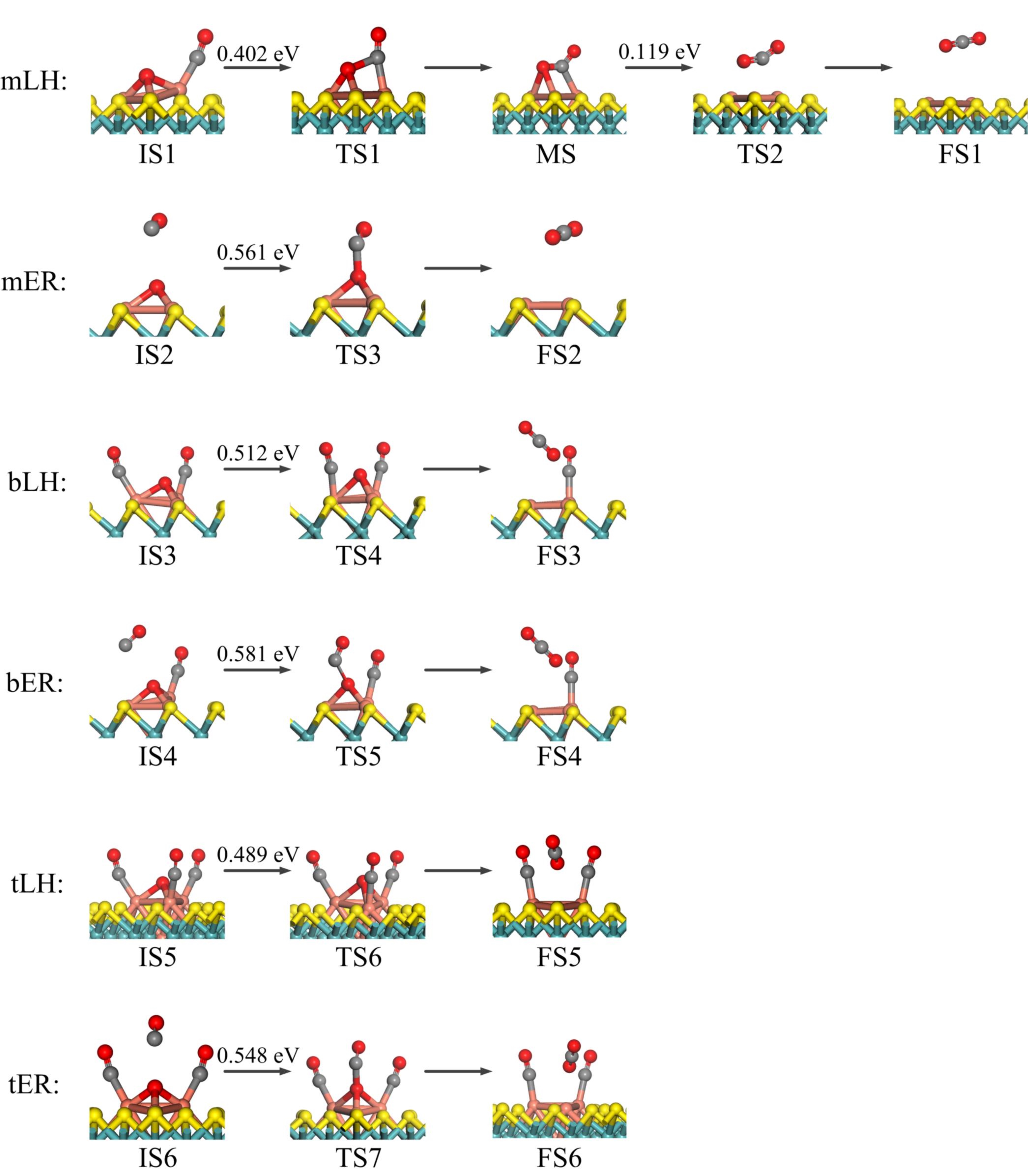
**

**Figure S3.** The reaction paths for the first CO_2_ release through LH mechanism (a) and ER mechanism (b). All energies are given with respect to the reference energy. The values in the figure indicated energy barriers.

**
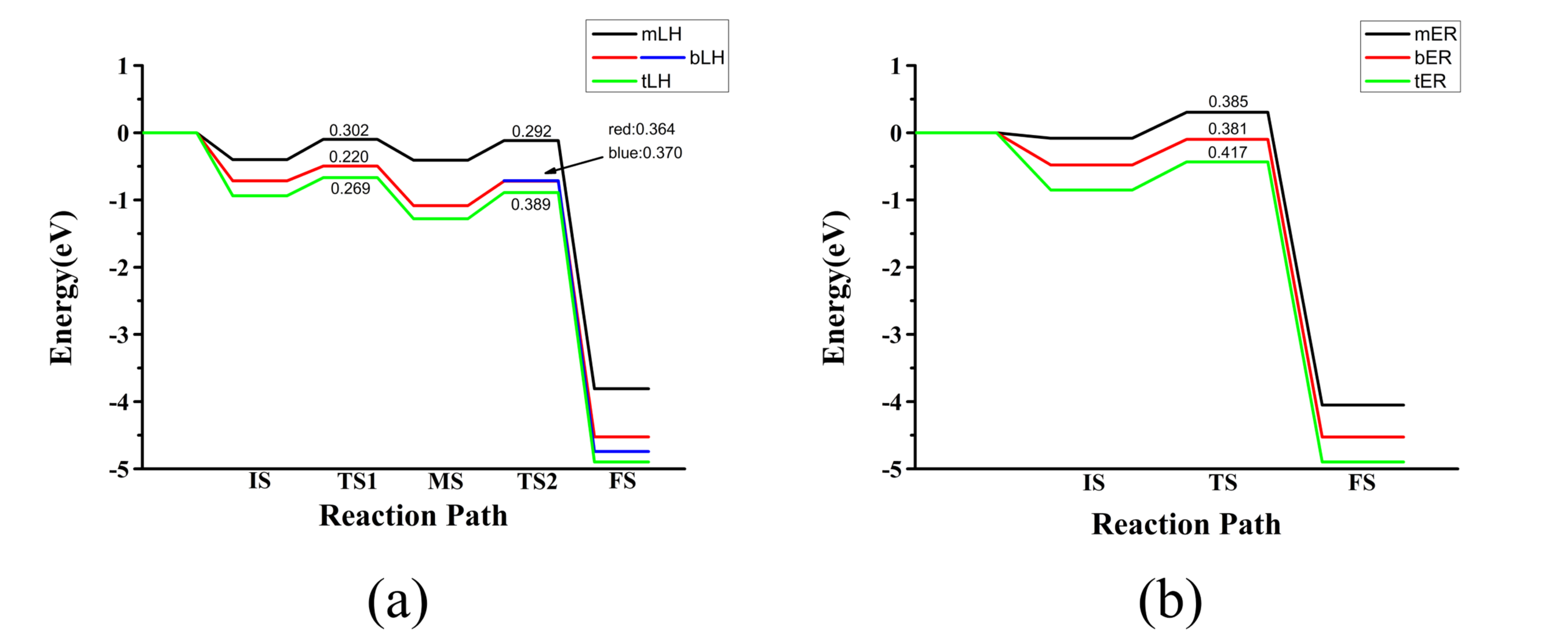
**

**Figure S4.** The reaction paths for the second CO_2_ release through LH mechanism (a) and ER mechanism (b). All energies are given with respect to the reference energy. The numbers in the figure indicated energy barriers.

**
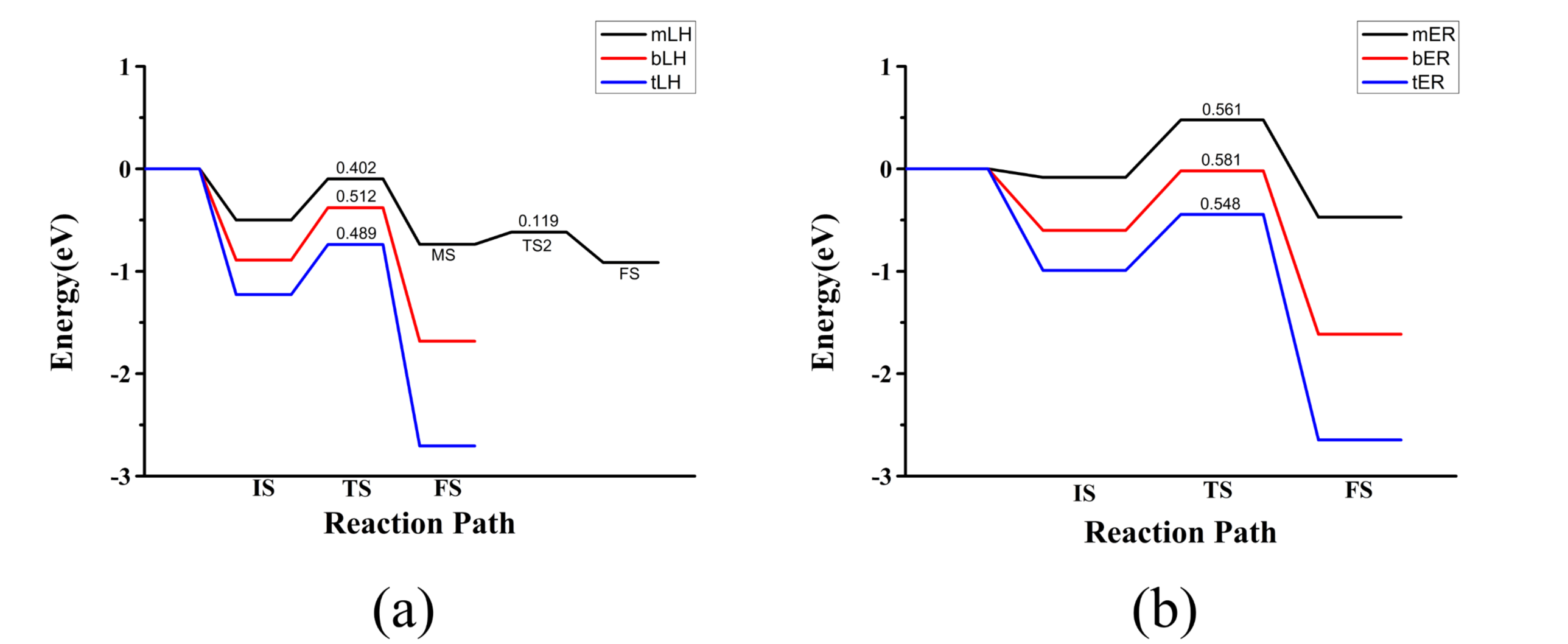
**

**Figure S5.**The dynamics process of Cu_4_ doped 3 × 3 monolayer MoS_2_ supercell at *T* = 500 K in the NVT ensemble (i.e., constant particle number, volume and temperature condition) has been carried out for 5 *ps* with the time step of 1 *fs*. Three structures from MD calculation are present in the figure.


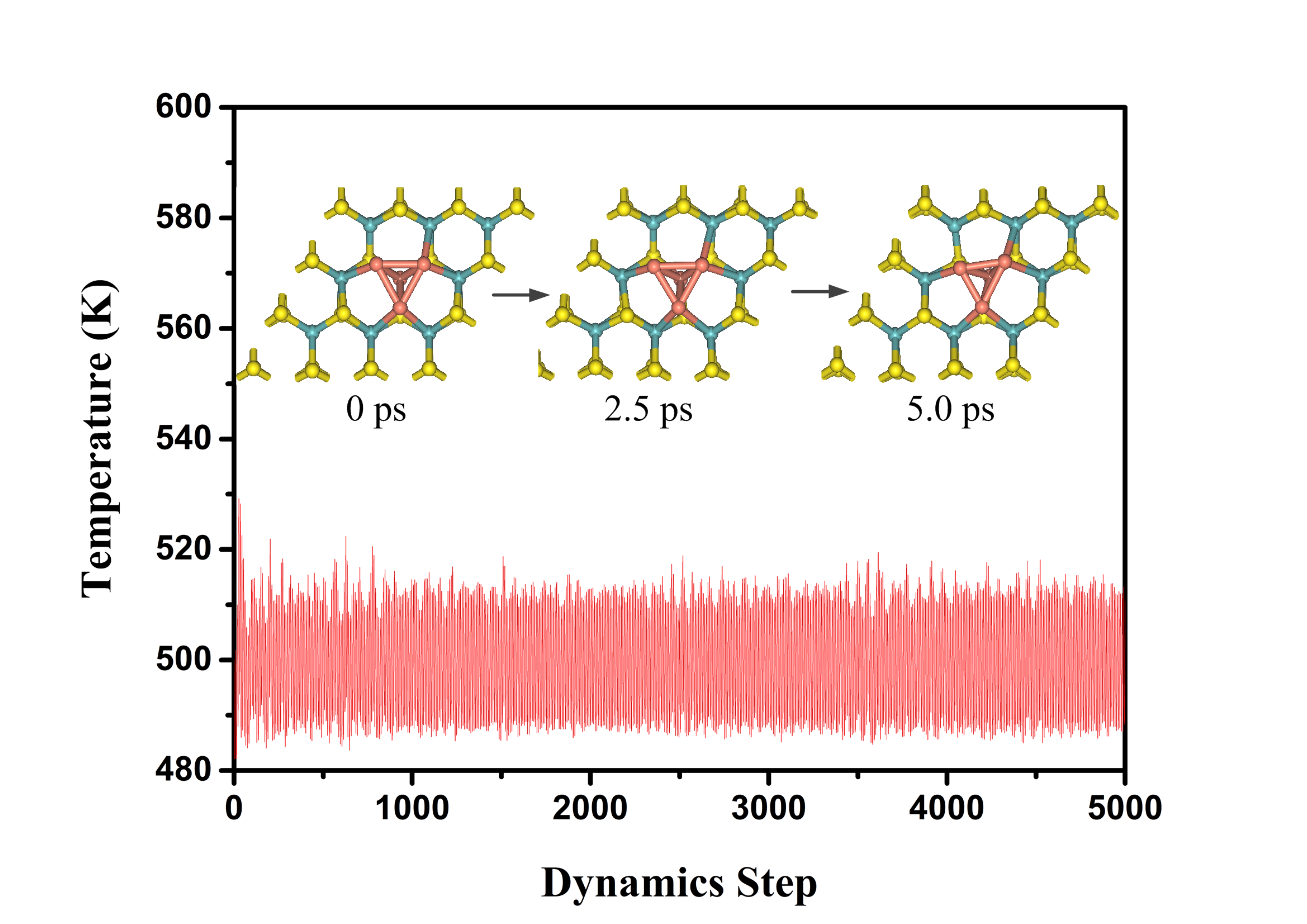


1. * Correspondence and requests for materials should be addressed to Q.J. (e-mail: jiangq@jlu.edu.cn). [↑](#footnote-ref-1)
